# Do core psychiatry trainee cognitive behaviour therapy cases meet training needs? An evaluation of core psychiatry trainee delivered CBT cases in Sheffield: implications for training and services

**DOI:** 10.1192/bjo.2021.804

**Published:** 2021-06-18

**Authors:** Dasal Abayaratne, Russell Birkett, John Davies

**Affiliations:** 1 Sheffield Health and Social Care NHS Foundation Trust; 2 University of Sheffield, Sheffield Health and Social Care NHS Foundation Trust

## Abstract

**Aims:**

This evaluation aims to understand if Cognitive Behaviour Therapy (CBT) cases for Core Psychiatry Trainees (CPTs) in Sheffield provide good training in therapy skills and if these can be integrated into general psychiatric practice.

**Background:**

Completion of psychotherapy cases part of the curriculum for CPTs, with cognitive behavior therapy being one of the common modalities used. Whilst there is evidence that trainees often provide competent therapy it is unclear what cases are appropriate and how these contribute to wider CPT learning objectives.

**Method:**

CPTs who had completed a clinical case in CBT at a tertiary psychotherapy service were identified. All were surveyed and patient demographics and outcomes also collated.

**Result:**

The results showed a significant impact on trainees understanding of CBT, applying theory to clinical context, and changed future practice. Despite being complex, 64% of patients needed no further therapy and 42% were discharged from mental health services.

**Conclusion:**

The evaluation demonstrates the positive outcomes for patients, trainees, future clinical practice, and a move towards collaboration as laid out in the Five-year forward view for mental health. This suggests that medical trainees have a valuable contribution, and role despite minimal experience in CBT.

